# The expression of CTLA-4 in hepatic alveolar echinococcosis patients and blocking CTLA-4 to reverse T cell exhaustion in *Echinococcus multilocularis*-infected mice

**DOI:** 10.3389/fimmu.2024.1358361

**Published:** 2024-03-28

**Authors:** Yuxuan Yang, Tana Wuren, Binjie Wu, Shilei Cheng, Haining Fan

**Affiliations:** ^1^ Research Center for High Altitude Medicine, Qinghai University, Key Laboratory of High Altitude Medicine (Ministry of Education), Key Laboratory of Application and Foundation for High Altitude Medicine Research in Qinghai Province (Qinghai-Utah Joint Research Key Lab for High Altitude Medicine), Laboratory for High Altitude Medicine of Qinghai Province, Xining, Qinghai, China; ^2^ Qinghai Research Key Laboratory for Echinococcosis, Qinghai University, Xining, Qinghai, China; ^3^ Department of Hepatopancreatobiliary Surgery, Affiliated Hospital of Qinghai University, Xining, Qinghai, China

**Keywords:** alveolar echinococcosis, *Echinococcus multilocularis*, immune microenvironment, cytotoxic T-lymphocyte antigen 4, T cell exhaustion

## Abstract

Alveolar echinococcosis (AE) is a zoonotic parasitic disease caused by the infection of *Echinococcus multilocularis* (*E. multilocularis*) larvae. Cytotoxic T-lymphocyte antigen 4 (CTLA-4) produces inhibitory signals and induces T cell exhaustion, thereby inhibiting the parasiticidal efficacy of the liver immune system. Therefore, the purpose of this study is to explore how T-cell exhaustion contributes to AE and whether blocking CTLA-4 could reverse T cell exhaustion. Here we discovered that the expression of CTLA-4 was increased in the infiltrating margin around the lesion of the liver from AE patients by using western blot and immunohistochemistry assay. Multiple fluorescence immunohistochemistry identified that CTLA-4 and CD4/CD8 molecules were co-localized. For *in vitro* experiments, it was found that the sustained stimulation of *E. multilocularis* antigen could induce T cell exhaustion, blocking CTLA-4-reversed T cell exhaustion. For *in vivo* experiments, the expression of CTLA-4 was increased in the liver of *E. multilocularis*-infected mice, and the CTLA-4 and CD4/CD8 molecules were co-localized. Flow cytometry analysis demonstrated that the percentages of both CD4^+^ T cells and CD8^+^ T cells in the liver and peripheral blood were significantly increased and induced T exhaustion. When the mice were treated with anti-CTLA-4 antibodies, the number and weight of the lesions decreased significantly. Meanwhile, the flow cytometry results suggested that blocking CTLA-4 could effectively reverse T cell exhaustion and reactivate immune function. Our work reveals that blocking CTLA-4 could effectively reverse the T cell exhaustion caused by *E. multilocularis* and could be used as a novel target for the treatment of AE.

## 1 Introduction

Echinococcosis, an ancient and harmful zoonotic, shows a worldwide distribution (1, 2). It has been reported that more than 10 species of *Echinococcus* have been identified, with four major pathogenic strains: *Echinococcus granulosus*, *Echinococcus multilocularis*, *Echinococcus ligarthrus*, and *Echinococcus vogeli* (3). There are two common clinical types: cystic echinococcosis (CE) caused by *Echinococcus granulosus* larvae infection and alveolar echinococcosis (AE) caused by *Echinococcus multilocularis* (*E. multilocularis*) larvae infection (4, 5). Human infection with echinococcosis is mainly through the fecal–oral route. When the eggs enter the alimentary tract, the eggs hatch into larvae in the duodenum and converge into the portal vein through the mesenteric vein. The larvae firstly enter the liver and lead to hepatic echinococcosis, so the morbidity of echinococcosis is the highest in the liver, which takes approximately 70%, and AE originates almost 100% in the liver (2, 6, 7). The initial symptoms are usually sub-clinical, and the lesions of AE and CE may not cause clinical symptoms in the first 10–15 years of infection. Therefore, when patients are diagnosed in the hospital, the disease often progresses to an advanced stage (8, 9). AE not only shows invasiveness in the liver but also involves adjacent organs through direct contact. Similar to hepatocellular carcinoma, AE can develop distant metastasis, which creates great difficulties in the treatment of AE and becomes prone to recurrence (10). If diagnosed AE patients do not receive effective treatment, the mortality in 10 years can be up to 90% (8, 11).

Pathological staining of liver specimens from hepatic AE patients revealed that an infiltrating margin was formed with a large number of macrophages and T cells as the predominant infiltrating cells, including some neutrophils and eosinophils. These immune cells and cytokines (TGF-β and IL-10) constituted the inflammatory microenvironment of AE patients (12).

The inflammatory microenvironment plays a crucial role in parasitism, invasion, metastasis, calcification, and necrosis, which may result in completely different clinical outcomes (13). On the one hand, the liver mediates the clearance of *E. multilocularis* by regulating metabolism and initiating immune responses. On the other hand, *E. multilocularis* induces immune suppression through secreted antigens (proteins and peptides secreted by *E. multilocularis* and metabolites contained in *E. multilocularis* vesicular fluid) to maintain long-term parasitism. Moreover, the specific immune response against AE is an important factor affecting the clinical outcome (14). Therefore, a full understanding of the molecular mechanisms of immunosuppression is crucial for restoring the host immune response against AE.

Co-inhibitory molecules have attracted great attention in immunology, such as cytotoxic T-lymphocyte antigen 4 (CTLA-4), programmed cell death 1 (PD-1), lymphocyte-activation gene 3 (LAG-3), and T cell immunoreceptor with immunoglobulin and immunoreceptor tyrosine-based inhibitory motif domain (TIGIT). It has been widely reported that the co-inhibitory molecules play a negative regulatory role by recognizing corresponding ligands and transmitting inhibitory signals to T cells (15, 16). In the process of chronic infection, although antigen-specific T cells initially acquire effector function, these effector function gradually diminished with prolonged antigen stimulation, and this dysfunction state represents a unique state, known as T cell exhaustion (17). T cell exhaustion usually shows a series of abnormal characteristics, such as progressive loss of effector function, continuous upregulation and co-expression of multiple inhibitory receptors, and immunometabolism disorders (18, 19). It was found that TIGIT expression was significantly increased in CD4^+^ T cells and CD8^+^ T cells in the peripheral blood and liver of hepatic AE patients, which induces T cell exhaustion and plays a key role in the immune escape of *E. multilocularis*. Blocking TIGIT in mice infected with *E. multilocularis* could reverse T cell exhaustion and inhibit the growth of lesions (20, 21).

CTLA-4, a member of the CD28 family, is a transmembrane receptor on T cells and is expressed on various types of T cells (22). CTLA-4 is structurally highly homologous to CD28, and both bind to ligands CD80 (B7-1) and CD86 (B7-2), which are located on antigen-presenting cells. CD28 binds to CD80/CD86 to provide co-stimulatory signals to activate T cells, whereas CTLA-4 has a significantly higher affinity for CD80/CD86 than CD28. When CTLA-4 competitively binds to CD80/CD86, it generates inhibitory signals to T cells and induces T cell exhaustion (23).

In summary, AE shows invasive growth, with advanced stage symptoms similar to hepatocellular carcinoma, which seriously threatens the physical and mental health of patients. However, there are still some deficiencies in the current diagnosis and treatment strategies, so it is urgent to explore new strategies. Defining the molecular mechanism of immunosuppression in the inflammatory microenvironment is expected to improve the clinical outcomes. T cell exhaustion is an important participant in hepatic immunosuppression, and several studies have demonstrated that CTLA-4 plays an important role in T cell exhaustion. Hence, we hope to investigate whether CTLA-4 mediates T cell exhaustion and make CTLA-4 a novel therapeutic target for reversing the immunosuppressive effect in AE patients.

## 2 Materials and methods

### 2.1 Experimental animals and human subjects

Specific pathogen-free male BALB/c mice (male, aged 4 weeks, 18–20 g) were purchased from Nanjing Qinglongshan Animal Breeding Base, Jiangning District, Nanjing, China. The mice were housed in a suitable environment, specific pathogen-free, with a temperature of 25°C and a 12-h light/dark cycle, and provided with rodent food and water. BALB/c mice were used to obtain splenocytes, construct an *E. multilocularis* infection model, and evaluate the therapeutic effect of anti-CTLA-4 antibody. All animal experiments were approved by the Affiliated Hospital of Qinghai University (P-SL-2019039), and all animals received humanitarian treatment.

The liver samples of AE patients were obtained from the Affiliated Hospital of Qinghai University, and all patients were pathologically diagnosed as hepatic AE. A total of 15 liver samples were collected from AE patients. The sample was divided into close to lesion tissue (CLT) containing an infiltrating margin (within 0.5 cm from the lesion) and distant to lesion tissue (DLT) containing liver parenchyma (more than 2 cm from the lesion) that does not contain the lesion. The liver samples of AE patients were used for hematoxylin and eosin (H&E) staining, Masson trichrome staining, immunohistochemistry staining, multiple fluorescence immunohistochemistry staining, and western blot. This study was approved by the Affiliated Hospital of Qinghai University (P-SL-2019039) in accordance with the Declaration of Helsinki and Istanbul, and all patients were given informed consent.

### 2.2 Isolation of *E. multilocularis* protoscoleces and acquisition of *E. multilocularis* antigen

*E. multilocularis* protoscoleces were isolated as described previously (24). *E. multilocularis* protoscoleces were obtained from Mongolian gerbils infected with *E. multilocularis* maintained in our laboratory. Mongolian gerbils were sacrificed, and the metacestode tissues were isolated from the abdominal cavity, cut into small pieces in pre-cooled physiological saline, filtered through 100- and 40-μm sterile filters to collect the protoscoleces, and washed continuously by using physiological saline in the process (the first washing of physiological saline was collected for use), and the protoscoleces of natural settlement were finally obtained. Viable protoscoleces were counted by using trypan blue and cultured in 1640 medium containing 15% fetal bovine serum. The protoscoleces were used to obtain *E. multilocularis* antigen and construct an *E. multilocularis* infection model.

For the *E. multilocularis* antigen, isolated viable protoscoleces were collected and suspended in previously collected physiological saline for washing. The protoscoleces were broken by using a tissue homogenizer and ultrasound pulse, and then it was centrifugated at 4°C and 12,000 rpm for 20 min, filtrated with sterile filter (0.22 μm), and stored at -80°C. *E. multilocularis* antigen was prepared to simulate splenocytes *in vitro*, inducing CTLA-4 expression and T cell exhaustion.

### 2.3 Purification and culture of splenocytes

Splenocytes were purified by referring to the previous method (25). The mice not infected with *E. multilocularis* were sacrificed, and the spleens were separated in a clean bench, washed with pre-cooled physiological saline, then cut into small pieces, and mechanically dispersed by using a sterile syringe plunger to force through a 70-μm cell strainer to obtain splenocytes. The erythrocytes were lysed with BD Pharm Lyse™ Lysing Buffer (BD Bioscience). The cells were re-suspended in 1640 medium containing 15% fetal bovine serum and cultured at 37°C in 5% CO_2_ incubator.

### 2.4 Multiple fluorescence immunohistochemistry of the liver in patients and mice infected with *E. multilocularis*

For multiple fluorescence immunohistochemistry, Liu et al. can be referred to (26), and a four-color multiple fluorescence immunohistochemistry staining kit (Absin, Shanghai, China) was used. Briefly, the slices were processed for heat-mediated antigen retrieval by using citric acid buffer. The sections were blocked with goat serum, and then primary antibody was added and incubated overnight at 4°C. On the next day, the sections were washed with TBST and incubated with a second antibody for 15 min, then washed with TBST, and incubated with tyramine signal amplification (TSA) monochrome fluorescent dye for 10 min, after which the above-mentioned steps were repeated. Finally, 4′6-diamino-2-phenylindole (DAPI) was used for nuclear staining. All images were acquired by using Nikon A1 plus a laser confocal microscope. The primary antibodies used for immunofluorescence were anti-CTLA4 antibody (CAL49; 1:500, Abcam), anti-CD4 antibody (EPR6855; 1:500, Abcam), anti-CD4 antibody (EPR19514; 1:1,000, Abcam), anti-CD8 alpha antibody (EPR21769; 1:2,000, Abcam), and anti-CD8 alpha antibody (EPR22483-288; 1:1,000, Abcam).

### 2.5 Mice infected with *E. multilocularis* and CTLA-4 blocking in mice infected with *E. multilocularis*

The viable *E. multilocularis* protoscoleces were collected and re-suspended with physiological saline. The mice were anesthetized with isoflurane and injected with 1,000 protoscoleces by *in situ* liver puncture injection. The mice in the sham operated group (control group) were injected with physiological saline.

For *in vivo* experiments, CTLA-4 blocking and albendazole (ABZ) treatment were started on the 8th week after infection. The mice in the CTLA-4 blocked group were given 200 ug [*In Vivo* Plus anti-mouse CTLA-4 (CD152), clone9H10, Bio X Cell] intraperitoneal injection each time (27), once every other day for 4 consecutive weeks. The mice in the ABZ treatment group received 100 mg/kg (Yuanye BioTechnology, Shanghai, China) (28) treatment once a day for 4 consecutive weeks. The mice in the IgG isotype antibody treatment group were intraperitoneally injected with 200 ug (*In Vivo* MAb polyclonal Syrian hamster IgG, Bio X Cell) each time once every other day for 4 consecutive weeks.

### 2.6 Determination of the *in vitro* CTLA-4 expression, T cell exhaustion, and blocking CTLA-4 reversal T cell exhaustion in BALB/c splenocyte culture by flow cytometry

For the detection of CTLA-4 expression and the determination of T cell exhaustion *in vitro*, anti-CD3/CD28 (*In Vivo* MAb anti-mouse CD28, clone 37.51, 10 ug/ml, Bio X Cell; *In Vivo* MAb anti-mouse CD3ϵ, clone 145-2C11, 10 ug/ml, Bio X Cell) were used to stimulate cells (29), and *E. multilocularis* antigen (Em-Ag, protein concentration 1 µg/mL) (30) was added to the co-culture for 72 h. The control group was cultured splenocytes in the absence of Em-Ag. The cells were re-suspended by medium and adjusted to 1 × 10^5^ per flow tube for flow cytometry. For the cell surface markers, surface marker flow cytometry antibodies are used to stain at 4°C for 30 min. For intracellular flow cytometry, the cells were cultured at 37°C for 4 h in the presence of BFA and PMA [BFA/monensin mixture (250×), MULTI SCIENCES, Hangzhou, China; PMA/ionomycin mixture (250×), MULTI SCIENCES, Hangzhou, China], stained with surface marker flow cytometry antibodies at 4°C for 30 minutes, then treated with BD Cytofix/Cytoperm™ Fixation/Permeabilization Kit (BD Bioscience) for 30 min, washed, and stained with intracellular flow cytometry antibodies at 4°C for 30 min. BD FACSCelesta™ Flow Cytometer (BD Bioscience) was used for data acquisition, and novoexpress1.5.6 software was used for data processing.

For the detection of anti-CTLA-4 reverse T cell exhaustion *in vitro*, T cell exhaustion was first induced and then treated with anti-CTLA-4 antibody [*In Vivo* Plus anti-mouse CTLA-4 (CD152), clone9H10, Bio X Cell] and homologous control IgG antibody (*In Vivo* MAb polyclonal Syrian hamster IgG, Bio X Cell) at 10 ug/ml for 72 h (31). The cells were adjusted to 1 × 10^5^ cells per flow tube for flow cytometry. The flow cytometry assay protocol was the same as described above. More details on the antibodies can be found in **Supplementary Table S1**.

### 2.7 Determination of the *in vivo* CTLA-4 expression, T cell exhaustion, and blocking CTLA-4 reversal T cell exhaustion in the blood cells and liver of BALB/c mice by flow cytometry

For the detection of CTLA-4 expression and T cell exhaustion *in vivo*, the mice were sacrificed at the 8th week of infection, and peripheral blood and the liver were collected. For peripheral blood samples, 100 ul peripheral blood samples were added to each flow tube. Erythrocytes were lysed with BD Pharm Lyse™ Lysing Buffer (BD Bioscience) at 4°C for 30 min and washed for staining. For the liver samples, the liver was prepared into a single cell suspension by enzymatic digestion and physical grinding method. Briefly, the liver was cut into small pieces and digested with type II collagenase for 30 min, and the digestive sample was dispersed by using a sterile syringe plunger to force through a 70-μm cell strainer to obtain a single-cell suspension. Then, the erythrocytes were lysed with BD Pharm Lyse™ Lysing Buffer (BD Bioscience) at 4°C for 30 min and washed for staining.

For the cell surface markers, surface marker flow cytometry antibodies are used to stain at 4°C for 30 min. For intracellular flow cytometry, the cells were cultured at 37°C for 4 h in the presence of BFA and PMA [BFA/Monensin Mixture (250×), MULTI SCIENCES, Hangzhou, China; PMA/ionomycin mixture (250×), MULTI SCIENCES, Hangzhou, China], stained with surface marker flow cytometry antibodies at 4°C for 30 min and then were treated with BD Cytofix/Cytoperm™ Fixation/Permeabilization Kit (BD Bioscience) for 30 min, washed, and stained with intracellular flow cytometry antibodies at 4°C for 30 min. BD FACSCelesta™ Flow Cytometer (BD Bioscience) was used for data acquisition, and novoexpress1.5.6 software was used for data processing.

For *in vivo* blocking CTLA-4 reversal T cell exhaustion assay, CTLA-4 blocking was performed as described previously. The mice were sacrificed, and peripheral blood and the liver were collected. Pretreatment of peripheral blood before staining and preparation of single-cell suspension of the liver were performed as described previously. The flow cytometry assay protocol was the same as described above. More details of antibodies can be found in **Supplementary Table S1**.

### 2.8 Statistical analysis

Statistical analysis was performed by using GraphPad Prism 8.0 software and plotted. The data are expressed as the mean ± standard deviation (SD). Student’s *t*-test was adopted in comparison between two groups and one-way ANOVA in multiple comparisons among groups of more than two. *P* values < 0.05 were considered significant.

## 3 Results

### 3.1 The liver pathology of AE patients and the expression of CTLA-4 is increased in the liver of AE patients

Firstly, in order to observe the histopathology of liver, H&E staining and Masson staining were performed on the biopsy. H&E staining showed that there were scattered vacuoles within the liver. Most vacuoles could be observed in the laminated layer and occasionally in the germinal layer. The infiltrating margin composed of various inflammatory cells was formed around the lesion, accompanied by tuberculous granuloma, and Masson staining revealed the presence of fibrosis in the vicinity of this area (**Supplementary Figures S1A, B**).

**Figure 1:**
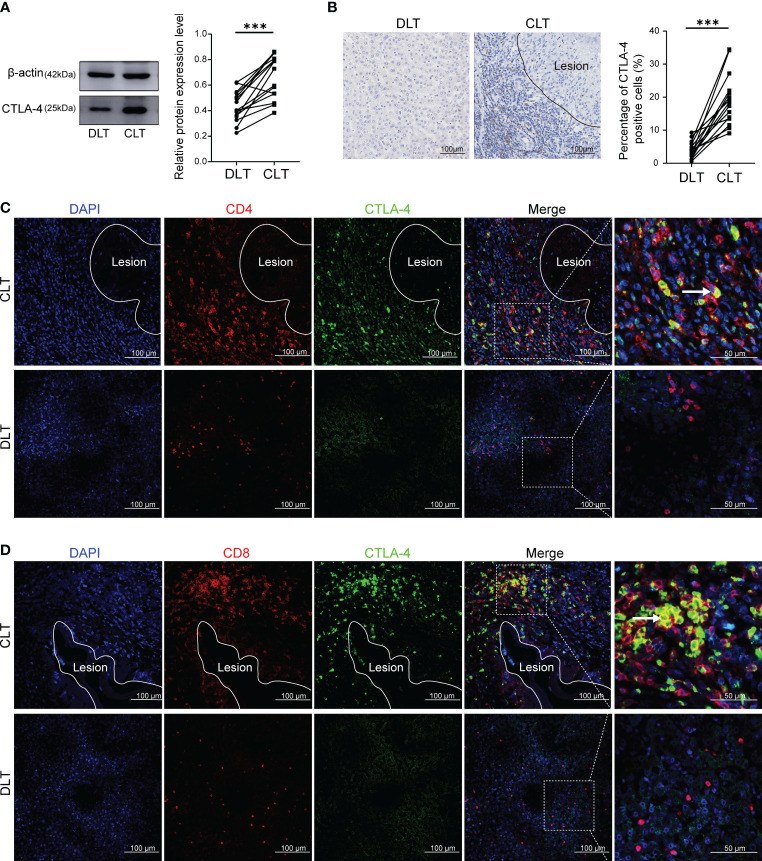
Expression of CTLA-4 in the liver of AE patients and its co-localization with CD4 and CD8 molecules. **(A)** The expression of CTLA-4 in CLT and DLT of liver AE patients was assayed by western blot, and the relative expression level was revealed (*n* = 15). **(B)** The expression of CTLA-4 in hepatic AE patients was detected by immunohistochemistry, and the percentage of CTLA-4 positive cells was indicated (*n* = 15). **(C)** Multiple fluorescence immunohistochemistry showed the co-localization of CD4 molecules and CTLA-4 in the CLT and the DLT of hepatic AE patients. The arrow indicates CD4^+^ CTLA-4^+^ T cells. Scale bar = 100 μm. **(D)** Multiple fluorescence immunohistochemistry showed the co-localization of CD8 molecules and CTLA-4 in the CLT and the DLT of hepatic AE patients. The arrow indicates CD8^+^ CTLA-4^+^ T cells. Scale bar = 100 μm. The data were presented as mean ± standard deviation(SD). ***P < 0.001. CLT, close to lesion tissue; DLT, distant to lesion tissue.

CTLA-4 acted as an inhibitory receptor and generates inhibitory signals on activated T cells, thereby inducing T cell exhaustion. Therefore, we examined the expression of CTLA-4 in the liver of AE patients. Western blot showed that the expression of CTLA-4 in the CLT was higher than that in the DLT (**Figure 1A**). In addition, immunohistochemistry staining of CTLA-4 was performed, which showed that, compared with liver parenchyma, the percentage of CTLA-4-positive cells was higher in the infiltrating margin (**Figure 1B**). It is suggested that the expression of CTLA-4 is mainly concentrated in inflammatory cells in the infiltrating margin around the lesion. To clarify the cell specificity of CTLA-4, multiple fluorescence immunohistochemistry staining was used to label CTLA-4, CD4, and CD8 molecules, which showed that co-localization of CTLA-4 with CD4 and CTLA-4 with CD8 molecules could be observed in the infiltrating margin of the CLT (**Figures 1C, D**), but no co-localization was observed in the DLT (**Figures 1C, D**). The above-mentioned results suggest that CD4^+^ T cells and CD8^+^ T cells expressed CTLA-4 in the infiltrating margin around the lesion, which provided the basis of the immune environment for T cell exhaustion. However, whether the increased expression of CTLA-4 on CD4^+^ T cells and CD8^+^ T cells could lead to T cell exhaustion would be further demonstrated by *in vitro* and *in vivo* experiments.

### 3.2 The effect of Em-Ag on CTLA-4 in mice splenocytes

**Figure 2:**
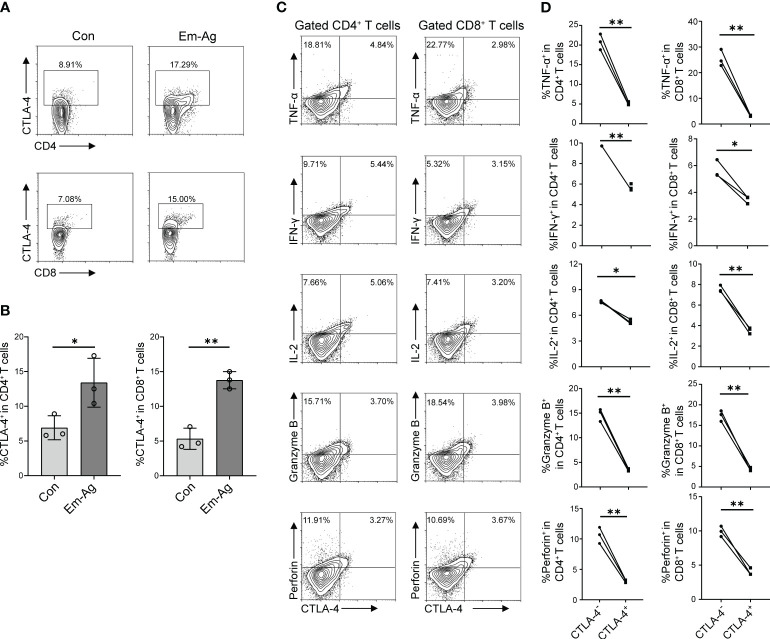
Em-Ag increased the expression of CTLA-4 and induced T cell exhaustion. **(A)** Representative flow cytometry of CD4^+^ CTLA-4^+^ T cells and CD8^+^ CTLA-4^+^ T cells after Em-Ag stimulation of mice splenocytes. **(B)** Percentage of CD4^+^ CTLA-4^+^ T cells and CD8^+^ CTLA-4^+^ T cells after Em-Ag stimulation of mice splenocytes (*n* = 3). **(C)** Representative flow cytometry plots of TNF-α, IFN-γ, IL-2, Granzyme B, and perforin production by CTLA-4^+^ CD4^+^ T cells compared with CTLA-4- CD4^+^ T cells or CTLA-4^+^ CD8^+^ T cells compared with CTLA-4- CD8^+^ T cells after Em-Ag stimulation of mice splenocytes. **(D)** Percentage of TNF-α, IFN-γ, IL-2, Granzyme B, and perforin production by CTLA-4^+^ CD4^+^ T cells compared with CTLA-4^-^ CD4^+^ T cells or CTLA-4^+^ CD8^+^ T cells compared with CTLA-4^-^ CD8^+^ T cells after Em-Ag stimulation of mice splenocytes (*n* = 3). The data are presented as mean ± SD. *P < 0.05, **P < 0.01. Con, control; Em-Ag, *E. multilocularis* antigen.

To investigate the effect of *E. multilocularis* on CTLA-4 expression, *in vitro* experiments were firstly performed. Since *E. multilocularis* was able to trigger immune responses by secreting various types of antigens and direct contact after infection, we added Em-Ag to stimulated T cells. After 72 h of culture, cells were extracted for flow cytometry detection. It was found that Em-Ag stimulation increased the percentage of CD4^+^CTLA-4^+^T cells and CD8^+^CTLA-4^+^T cells in mice splenocytes (**Figures 2A, B**), which indicated that *E. multilocularis* continuously stimulated T cells to induce the increase of CTLA-4.

To explore whether the increase of CTLA-4 in CD4^+^ T cells and CD8^+^ T cells induced T cell exhaustion, Em-Ag was added to mice splenocytes and anti-CD3 antibody and anti-CD28 antibody were used to stimulate T cells. After 72 h of culture, cells were collected and cytokines (IL-2, IFN-γ, TNF-α, Granzyme B, and perforin) were assayed by flow cytometry to determine whether the increase of CTLA-4 would cause T cell exhaustion. The results showed that CTLA-4^-^CD4^+^ T cells and CTLA-4^-^CD8^+^ T cells produced more cytokines compared with CTLA-4^+^CD4^+^ T cells and CTLA-4^+^CD8^+^ T cells, respectively (**Figures 2C, D**). So far, the *in vitro* experiments have demonstrated that *E. multilocularis* infection continuously stimulated CD4^+^ T cells and CD8^+^ T cells, leading to an increase in CTLA-4 and thereby inducing CD4^+^ T cell and CD8^+^ T cell exhaustion.

### 3.3 Blocking CTLA-4 reverses the CD4^+^ T cell and CD8^+^ T cell exhaustion in mice splenocytes

**Figure 3:**
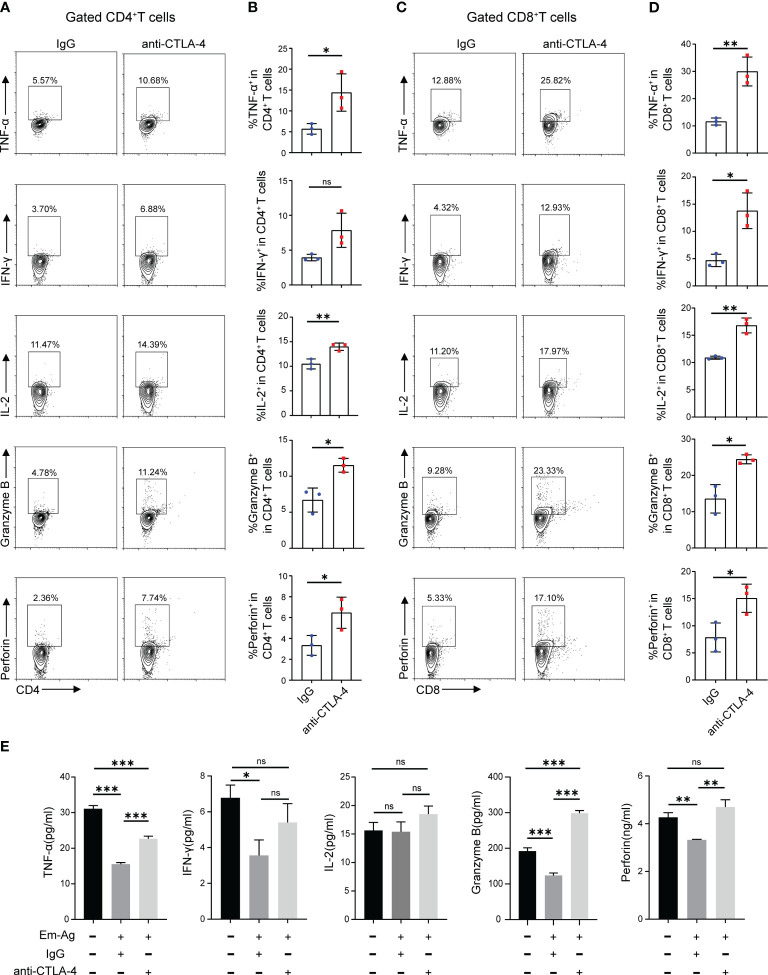
Blocking CTLA-4 reversed the CD4^+^ T cell and CD8^+^ T cell exhaustion in mice splenocytes. **(A)** Representative flow cytometry plots of TNF-α, IFN-γ, IL-2, Granzyme B, and perforin expression on mice spleen CD4^+^ T cells in the presence of IgG or anti-CTLA-4 antibody. **(B)** Percentage of TNF-α, IFN-γ, IL-2, Granzyme B, and perforin expression on mice spleen CD4^+^ T cells in the presence of IgG or anti-CTLA-4 antibody. **(C)** Representative flow cytometry plots of TNF-α, IFN-γ, IL-2, Granzyme B, and perforin expression on mice spleen CD8^+^ T cells in the presence of IgG or anti-CTLA-4 antibody. **(D)** Percentage of TNF-α, IFN-γ, IL-2, Granzyme B, and perforin expression on mice spleen CD8^+^ T cells in the presence of IgG or anti-CTLA-4 antibody. **(E)** The levels of TNF-α, IFN-γ, IL-2, Granzyme B, and perforin in the cell medium of three different groups (normal mice splenocytes, Em-Ag-stimulated and IgG-treated mice splenocytes, and Em-Ag-stimulated and anti-CTLA-4 antibody-treated mice splenocytes) were assayed by ELISA. “+”, treated; “-”, untreated. The data were presented as mean ± SD. *P < 0.05; **P < 0.01; ***P < 0.001; no significant difference (ns) *P* > 0.05.

The result has demonstrated that the increased expression of CTLA-4 induced CD4^+^ T cell and CD8^+^ T cell exhaustion. What to be found next was to explore whether blocking CTLA-4 could reverse the T cell exhaustion and reactivate immune function. Therefore, Em-Ag was added to induce CD4^+^ T cell and CD8^+^ T cell exhaustion. Afterward, the mice splenocytes were treated with anti-CTLA-4 antibody and isotype IgG antibody respectively for 72 h, and the cells were collected for flow cytometry. The results showed that, compared to the IgG treatment group, the percentages of CD4^+^ IL-2^+^ T cells, CD4^+^ TNF-α^+^ T cells, CD4^+^ Granzyme B^+^ T cells, and CD4^+^ perforin^+^ T cells in the anti-CTLA-4 antibody treatment group were increased, while the percentage of CD4^+^ IFN-γ^+^ T cells did not show a significantly difference between the two groups (**Figures 3A, B**), whereas in the detection of CD8^+^ T cells it was found that the percentages of CD8^+^ IL-2^+^ T cells, CD8^+^ TNF-α^+^ T cells, CD8^+^ IFN-γ^+^ T cells, CD8^+^ Granzyme B^+^ T cells, and CD8^+^ perforin^+^ T cells of anti-CTLA-4 antibody treatment group were all higher than that of the IgG antibody treatment group (**Figures 3C, D**). In addition, the culture medium of each group was also collected for enzyme-linked immunosorbent assay (ELISA). The results showed that, compared with the IgG treatment group, the contents of TNF-α, Granzyme B, and perforin in the culture medium of the anti-CTLA-4 antibody treatment group were increased (**Figure 3E**). The above-mentioned results suggested that blocking CTLA-4 could effectively reverse the CD4^+^ T cell and CD8^+^T cell exhaustion and contribute to restore the immune function.

### 3.4 *E. multilocularis* infection leads to an increased expression of CTLA-4 in CD4^+^ T cells and CD8^+^T cells in the liver and peripheral blood of mice

**Figure 4:**
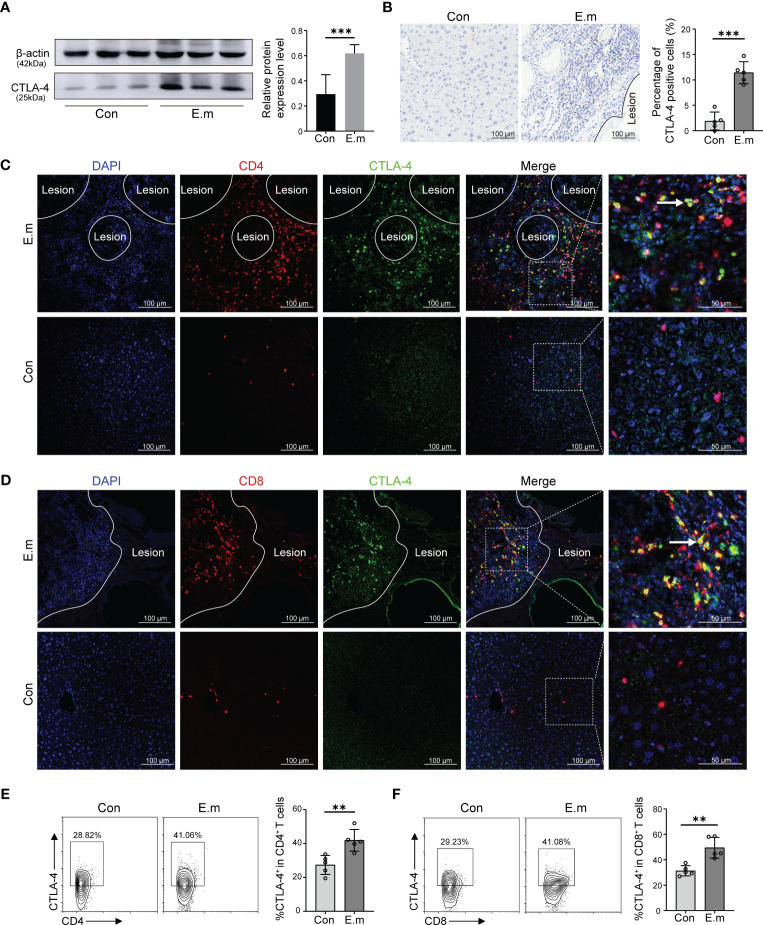
Effect of *E. multilocularis* infection on the expression of CTLA-4 in the liver of mice. **(A)** The expression of CTLA-4 in the liver of mice infected with *E. multilocularis* or control group was assayed by western blot, and the relative expression level was revealed (*n* = 5). **(B)** The expression of CTLA-4 in the mice liver of mice infected with *E. multilocularis* or control group was detected by immunohistochemistry, and the percentage of CTLA-4-positive cells was indicated (*n* = 5). **(C)** Multiple fluorescence immunohistochemistry showed the co-localization of CD4 molecules and CTLA-4 in the infiltrating margin of mice infected with *E. multilocularis* and the liver of control group. The arrow indicates CD4^+^ CTLA-4^+^ T cells. Scale bar = 100 μm. **(D)** Multiple fluorescence immunohistochemistry showed the co-localization of CD8 molecules and CTLA-4 in the infiltrating margin of mice infected with *E. multilocularis* and the liver of the control group. The arrow indicates CD8^+^ CTLA-4^+^ T cells. Scale bar = 100 μm. **(E)** Representative flow cytometry plots and percentage of CD4^+^ CTLA-4^+^ T cells in the liver of mice infected with *E. multilocularis* or control group (*n* = 5). **(F)** Representative flow cytometry plots and percentage of CD8^+^ CTLA-4^+^ T cells in the liver of mice infected with *E. multilocularis* or control group (*n* = 5). The data were presented as mean ± SD. **P < 0.01, ***P < 0.001. Con, control; E.m, *E. multilocularis*.

Although it has been demonstrated that *E. multilocularis* lead to T cell exhaustion and blocking CTLA-4 can reverse T cells exhaustion *in vitro*, cytological experiments do not fully represent the *in vivo* environment. To represent the *in vivo* infection of *E. multilocularis*, a model of mice infected with *E. multilocularis* larvae was constructed, which was divided into sham operated and *E. multilocularis* infection groups. Firstly, we observed the histopathology of infected mice through H&E and Masson staining. Similar to the AE patients, it can be observed that there were scattered vacuoles within the liver, the formation of infiltrating margin consisting of inflammatory cells around the lesion, and fibrosis in the vicinity of this area (**Supplementary Figures S1C, D**). The result of Western blot showed that the expression of CTLA-4 in the liver of infected mice was higher than that of sham operated mice (**Figure 4A**). Further immunohistochemistry staining revealed that the expression of CTLA-4 in the infiltrating margin of infected mice was significantly higher than that in the liver of sham operated mice (**Figure 4B**), indicating that *E. multilocularis* infection can cause an increase of CTLA-4 in the liver, especially in the infiltrating margin around the lesion.

To investigate the cell specificity of CTLA-4, we conducted multiple fluorescence immunohistochemistry staining, and the results showed that, similar to AE patients, the co-localization of CTLA-4 with CD4 and CTLA-4 with CD8 molecules can be observed in the infiltrating margin of infected mice (**Figures 4C, D**), but no co-localization was observed in the liver of the control group (**Figures 4C, D**). Subsequently, the results of flow cytometry showed that the percent of CD4^+^CTLA-4^+^T cells and CD8^+^CTLA-4^+^T cells in the liver of infected mice was higher than that of sham operated mice (**Figures 4E, F**), which more precisely showed that *E. multilocularis* infection led to the increase of CTLA-4 in CD4^+^ T cells and CD8^+^T cells.

When the liver is infected with *E. multilocularis*, the antigens secreted by *E. multilocularis* and the immune cells involved in anti-*E. multilocularis* have systemic effects throughout the blood circulation, which may cause systemic T cell exhaustion. Therefore, we assayed CD4^+^ T cells and CD8^+^T cells not only in the liver but also in the peripheral blood. The results showed that CD4^+^CTLA-4^+^T cells and CD8^+^CTLA-4^+^T cells of infected mice were higher than those of sham operated mice (**Supplementary Figure S2A, B**), indicating that *E. multilocularis* infection caused systemic changes in immune cells.

### 3.5 *E. multilocularis* infection leads to CD4^+^ T cell and CD8^+^ T cell exhaustion in the liver and peripheral blood of mice

**Figure 5:**
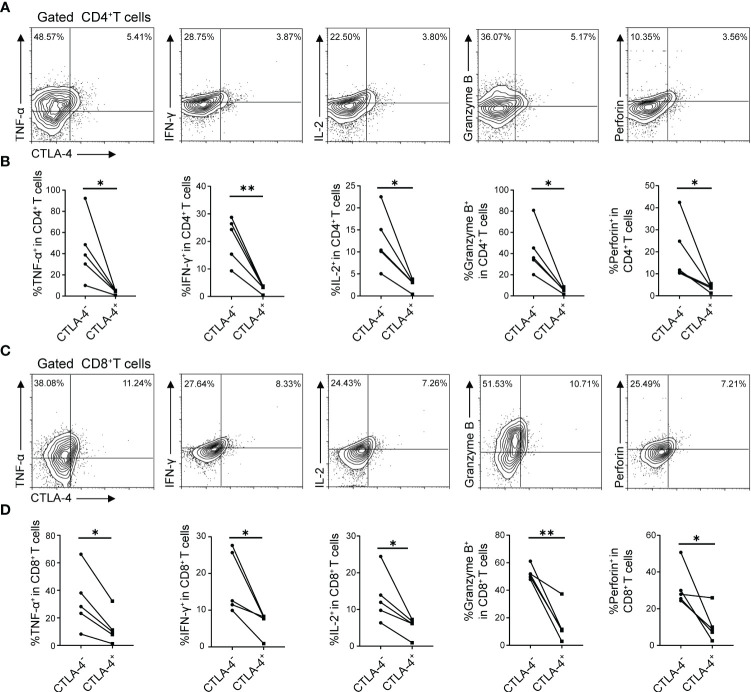
*E. multilocularis* infection led to CD4^+^ T cell and CD8^+^ T cell exhaustion in the liver of mice. **(A)** Representative flow cytometry plots of TNF-α, IFN-γ, IL-2, Granzyme B, and perforin production by CD4^+^ T cells in the liver of mice infected with *E. multilocularis* that did or did not express CTLA-4. **(B)** Percentage of TNF-α, IFN-γ, IL-2, Granzyme B, and perforin production by CD4^+^ T cells in the liver of mice infected with *E. multilocularis* that did or did not express CTLA-4 (*n* = 5). **(C)** Representative flow cytometry plots of TNF-α, IFN-γ, IL-2, Granzyme B, and perforin production by CD8^+^ T cells in the liver of mice infected with *E. multilocularis* that did or did not express CTLA-4. **(D)** Percentage of TNF-α, IFN-γ, IL-2, Granzyme B, and perforin production by CD8^+^ T cells in the liver of mice infected with *E. multilocularis* that did or did not express CTLA-4 (n = 5). The data were presented as mean ± SD. *P < 0.05, **P < 0.01.

In the previous part of experiments, it has been demonstrated that *E. multilocularis* infection led to the increase of CTLA-4 in CD4^+^ T cells and CD8^+^ T cells, and the increase of CTLA-4 might induce T cell exhaustion *in vitro*. Therefore, what to be investigated next was whether CTLA-4 could induce T cell exhaustion *in vivo*. Flow cytometry was used to assay the cytokine production (IL-2, IFN-γ, TNF-α, Granzyme B, and perforin) of CD4^+^ T cells and CD8^+^ T cells in the liver and peripheral blood, respectively. The results showed that, among liver CD4^+^ T cells and CD8^+^ T cells, CTLA-4^-^ cells produced more cytokines than CTLA-4^+^ cells, respectively (**Figures 5A-D**). Similar results showed that, among peripheral blood CD4^+^ T cells and CD8^+^ T cells, CTLA-4^-^ cells produced more cytokines than CTLA-4^+^ cells, respectively (**Supplementary Figures S2C, D**). The results indicated that *E. multilocularis* infection caused not only CD4^+^ T cell and CD8^+^T cell exhaustion in the liver but also CD4^+^ T cell and CD8^+^T cell exhaustion in peripheral blood, which might be related to chronic infection in AE patients.

### 3.6 Blocking CTLA-4 treated *E. multilocularis* infection in mice by reversing T cell exhaustion

Chronic infection with *E. multilocularis* leads to an increased expression of CTLA-4 in CD4^+^ T cells and CD8^+^ T cells, which induces T cell exhaustion, resulting in immunosuppression in mice. Can blocking CTLA-4 reverse T cell exhaustion and reactivate immune function, thus treating the *E. multilocularis* infection?

**Figure 6:**
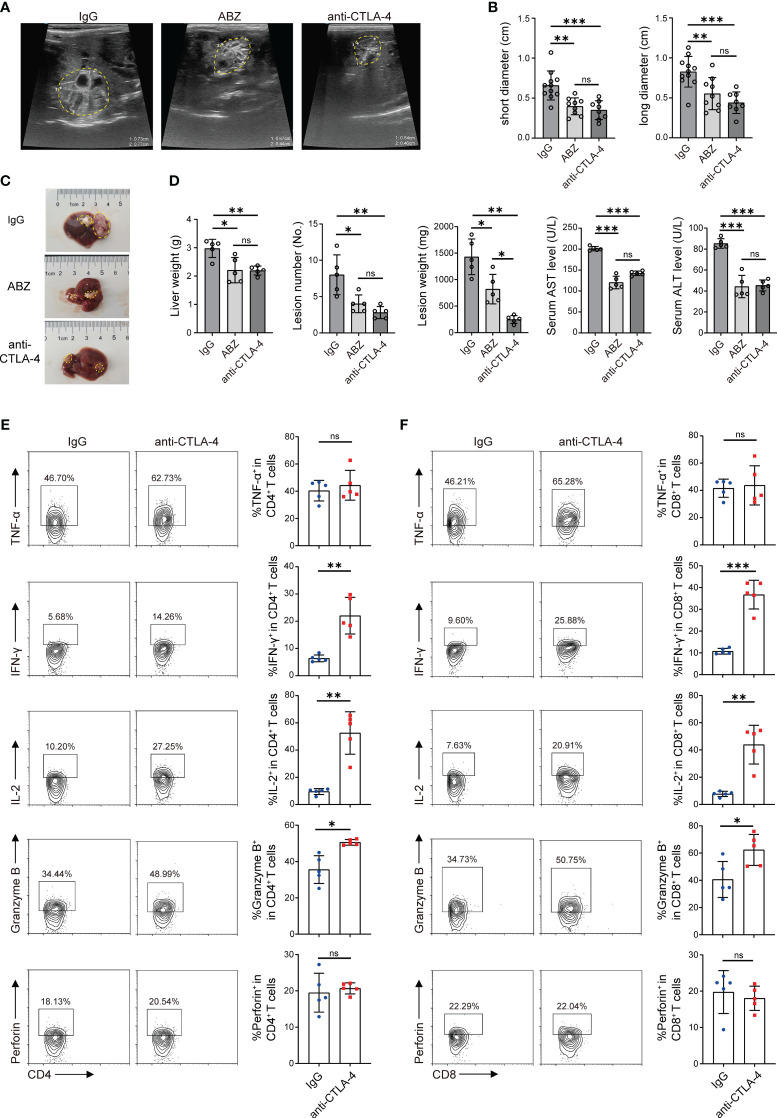
Blocking CTLA-4 reversed T cell exhaustion and treated *E multilocularis* infection in mice. **(A)** Representative ultrasound images of liver lesion tissues in mice treated with ABZ, anti-CTLA-4, and IgG, and lesion tissues were manually encircled with dashed yellow lines (*n* = 5). **(B)** The long diameters and short diameters of liver lesions in ABZ-, anti-CTLA-4-, and IgG-treated mice were measured by ultrasound. **(C)** Gross morphology of liver lesions in mice treated with ABZ, anti-CTLA-4, and IgG, and lesion tissues were manually encircled with dashed yellow lines (*n* = 5). **(D)** Liver weight, lesion number, and lesion weight of ABZ-, anti-CTLA-4-, and IgG-treated mice. The levels of AST and ALT in serum were detected (*n* = 5). **(E)** Representative flow cytometry plots and percentage of TNF-α, IFN-γ, IL-2, Granzyme B, and perforin expression in liver CD4+ T cells of mice treated with anti-CTLA-4 antibody and IgG antibody (*n* = 5). **(F)** Representative flow cytometry plots and percentage of TNF-α, IFN-γ, IL-2, Granzyme B, and perforin expression in liver CD8^+^ T cells of mice treated with anti-CTLA-4 antibody and IgG antibody (n = 5). The data were presented as mean ± SD. *P < 0.05; **P < 0.01; ***P < 0.001; no significant difference (ns) *P* > 0.05.

At the 12th week, the growth of lesions in mice livers was detected by ultrasound, and then all mice were sacrificed. Firstly, the size of the lesions in each group was initially evaluated by using a small animal ultrasound. Long diameters and short diameters of each observable lesion were used as evaluation indexes. The results showed that the lesions in the ABZ treatment group and anti-CTLA-4 antibody treatment group were smaller than those in the IgG antibody treatment group in terms of both long diameters and short diameters, but no significant differences were observed between the ABZ treatment group and the anti-CTLA-4 antibody treatment group (**Figures 6A, B**). Subsequently, the livers of each group were photographed and recorded (**Figure 6C**); the weight of the liver and the weight and number of lesions in each group were measured. The results showed that the liver weight, lesion weight, and lesion number in the ABZ treatment group and anti-CTLA-4 antibody treatment group were lower than those in the IgG treatment group, and the lesion weight of the anti-CTLA-4 antibody treatment group was lower than that in the IgG antibody treatment group, but there was no significant difference in liver weight and lesion number between the IgG antibody treatment group and the anti-CTLA-4 antibody treatment group (**Figure 6D**). In addition, the alanine aminotransferase (ALT) and aspartate aminotransferase (AST) of each group were assayed, and it was found that the ALT and AST of the ABZ treatment group and anti-CTLA-4 antibody treatment group were lower than those of the IgG antibody treatment group, but no significant differences were found between the ABZ treatment group and the anti-CTLA-4 antibody treatment group (**Figure 6D**). Finally, flow cytometry was used to assay the cytokines of CD4^+^ T cells and CD8^+^ T cells in the liver and peripheral blood. We found that, among the CD4^+^ T cells in the liver, the percentage of CD4^+^ IL-2^+^, CD4^+^ IFN-γ^+^, and CD4^+^ Granzyme B^+^ cells in the anti-CTLA-4 antibody treatment group was higher than in the IgG antibody treatment group, while the percentage of CD4^+^ TNF-α^+^, and CD4^+^ perforin^+^ cells was not significantly different between the two groups (**Figure 6E**). Similar results showed that, among the CD8^+^ T cells in the liver, the percentage of CD8^+^ IL-2^+^, CD8^+^ IFN-γ^+^, and CD8^+^ Granzyme B^+^ cells in the anti-CTLA-4 antibody treatment group was higher than those in the IgG antibody treatment group, while the percentage of CD8^+^ TNF-α^+^ and CD8^+^ perforin^+^ cells was not significantly different between the two groups (**Figure 6F**). However, for T cells in peripheral blood, among the CD4^+^ T cells, the percentage of CD4^+^ perforin^+^, CD4^+^ IL-2^+^, CD4^+^ IFN-γ^+^, and CD4^+^ Granzyme B^+^ cells in the anti-CTLA-4 antibody treatment group was higher than those in the IgG antibody treatment group, while the percentage of CD4^+^ TNF-α^+^ cells was not significantly different between the two groups (**Supplementary Figures S3A, B**). Similar results showed that, among the CD8^+^ T cells, the percentage of CD8^+^ perforin^+^, CD8^+^ IL-2^+^, CD8^+^ IFN-γ^+^, and CD8^+^ Granzyme B^+^ cells in the anti-CTLA-4 antibody treatment group was higher than those of the IgG antibody treatment group, while the percentage of CD8^+^ TNF-α^+^ cells was not significantly different between the two groups (**Supplementary Figures S3C, D**). Overall, the results suggested that blocking CTLA-4 successfully reverses the CD4^+^ T cell and CD8^+^ T cell exhaustion mice and reactivates immune function, thus playing a positive therapeutic role in mice infected with *E. multilocularis.*

## 4 Discussion

Echinococcosis remains a global public health problem, causing great harm to patients both physically and mentally and resulting in a series of social problems as well as significant economic losses (32, 33). Currently, the treatment strategy for hepatic AE is through surgery, intervention, and chemotherapy to achieve the purpose of eradicating symptomatic response, prolonging patient’s life, and improving the quality of life (6). However, due to the characteristics of recurrence and metastasis as well as poor absorption and utilization of ABZ (34), it is urgent to explore novel treatment strategies to make up for the inadequacies.

Tumor immunotherapy targets the immunosuppressive microenvironment, such as the tumor microenvironment (35). One of the main roles of the tumor microenvironment is to form an immunosuppressive environment around the tumor, weaken the killing effect of immune cells, and cause immune escape (36). AE is similar to “hepatocellular carcinoma” and exhibits invasive growth. Through pathological staining, we found that the infiltrating margin around the lesion was infiltrated by immune cells and formed an inflammatory microenvironment similar to hepatocellular carcinoma. That plays a crucial role in the clinical outcomes of AE patients.

T cell exhaustion is defined as a progressive dysfunction of T cell, which is mainly related to the continuous stimulation by antigens, and can be characterized by the expression or co-expression of inhibitory molecules (such as CTLA-4, PD-1, LAG-3, and Tim3), the decreased secretion of cytokines and effector factors, the altered expression and function of some transcription factors, and disturbance of immune metabolism (37, 38). Previous studies have shown that the immune response against echinococcosis is dominated by T cell-mediated cellular immunity (39). Therefore, we would like to examine the functional response to *E. multilocularis* infection. Western blot was used to assay the expression of CTLA-4 in the liver of AE patients, and it was found that CTLA-4 is increased in the CLT of AE patients. Immunohistochemistry staining showed that the infiltrating margin was the main region of increased CTLA-4 expression. Furthermore, multiple fluorescence immunohistochemistry staining revealed the co-localization of CD4/CD8 molecules and CTLA-4 in the infiltrating margin, which suggests that CD4^+^ T cells and CD8^+^ T cells in AE patients have an immune environment for T cell exhaustion.

The metabolites of *E. multilocularis* induce immune regulation in the host (40). To explore whether *E. multilocularis* infection induces CD4^+^ T cell and CD8^+^ T cell exhaustion, we first performed *in vitro* experiments. Studies have shown that E. multilocularis vesicular fluid induces the expression of immune checkpoints PD-1, CTLA-4, LAG-3, and TIM-3 on natural killer (NK) cells *in vitro* (30). Splenocytes were isolated from healthy mice and added with Em-Ag to stimulate T cells. The flow cytometry results showed that Em-Ag stimulation increased the percentage of CD4^+^ CTLA-4^+^ T cells and CD8^+^ CTLA-4^+^ T cells in splenocytes. Subsequently, CD4^+^ T cell and CD8^+^ T cell exhaustion was proved by flow cytometry. It was then demonstrated *in vitro* that blocking CTLA-4 reversed CD4^+^ T cell and CD8^+^ T cell exhaustion in mice, thereby reactivating immune functions.

AE and CE are common clinical types. Compared to AE, CE has a complete capsule, and the laminated layer of CE can provide physical and biological protection (41–43). It has been found that T cell-mediated immunity is impaired in the liver of CE patients (44). Currently, the treatment of CE has better efficacy, and the mortality rate caused by CE is much lower than that of AE (8). AE does not have a complete capsule, exhibits invasive growth, and has a unique immune microenvironment, which plays an important role in immune escape (12). Multiple studies have demonstrated that *E. multilocularis* infection induces the expression of co-inhibitory molecules, resulting in immunosuppression of the host. It has been found that the PD-1/PD-L1 pathway is over-expressed in mice infected with *E. multilocularis* (45). Blocking PD-L1 can enhance the host Th1 immune response and inhibit regulator T cell function at the early stage of infection as well as inhibit Th2 immune response at the advanced stage of infection (40). In addition, the number of TIGIT^+^ NK cells significantly increased in the infiltrating margin and peripheral blood of mice infected with *E. multilocularis*, leading to NK cell exhaustion. Knocking out TIGIT can reverse NK cell exhaustion and increase IFN-γ, which inhibits the growth of lesions and protects the liver (21). The mice were divided into sham operation and infection groups. Western blot and immunohistochemistry staining showed that CTLA-4 expression in the liver of infected mice was higher than that of sham operation mice, especially in the infiltrating margin around the lesion. The flow cytometry results precisely showed that *E. multilocularis* infection led to the increase of CTLA-4 on CD4^+^ T cells and CD8^+^ T cells in the liver.

Human immune organs are mainly divided into central immune organs, peripheral immune organs, and some organs/tissues with immune functions. Liver, as an important organ with complex structure and function, not only has functions such as digestion and detoxification but also is an important participant in immunity (46). It contains a variety of immune cells and cytokines. Antigens secreted by *E. multilocularis* and immune cells in the liver communicate with other tissues and organs through blood and lymph (43), which may have an impact on immune cells in other tissues and organs. In addition, circulating immune cells throughout the body trigger a systemic immune response of the host (12, 47, 48). These factors may cause systemic T cell exhaustion. We found that the percentage of CD4^+^ CTLA-4^+^ T cells and CD8^+^ CTLA-4^+^ T cells in the blood of infected mice was higher than that of sham operated mice, which indicated that *E. multilocularis* infection not only affects the immune microenvironment around the lesion but also has the ability to cause systemic changes. Afterward, we found that *E. multilocularis* infection induced CD4^+^T cell and CD8^+^T cell exhaustion by increasing CTLA-4 expression in the liver and peripheral blood.

So, can blocking CTLA-4 successfully reverse T cell exhaustion in mice and play a positive role in the treatment of *E. multilocularis* infection? Thus, anti-CTLA-4 antibody was used *in vivo*, and the curative effect was compared with ABZ (49). We found that there was no significant difference in liver weight and the number of lesions between the anti-CTLA-4 antibody treatment group and the ABZ treatment group, but the lesion weight of the anti-CTLA-4 antibody treatment group was lower than that of the ABZ treatment group.

The recommended chemotherapy regimens rely on ABZ, but the curative effect is not satisfactory because of the low utilization rate, the need for long-term administration, poor compliance, and the fact that ABZ can only inhibit the growth of parasites rather than kill parasites (50). In contrast, anti-CTLA-4 therapy, by reversing T cell exhaustion *in vivo* and reactivating immune function, directly kills parasites, and anti-CTLA-4 therapy has been approved for clinical therapy, with sufficient safety profile. Although anti-CTLA-4 therapy is mostly used in combination with other immune checkpoint therapies, for example, after hepatitis C infection, the expression of PD-1 and CTLA-4 on CD8^+^ T cells in peripheral blood increases, inducing T cell exhaustion, and blocking PD-1 or CTLA-4 alone fails to obtain a satisfactory curative effect, and only the combination of PD-1 and CTLA-4 was able to reverse T cell dysfunction (51, 52). However, studies in helminth has revealed that immune checkpoints have different effects. CTLA-4 has a profound effect on both CD4^+^ T cells and CD8^+^ T cells, while PD-1 has a primary effect on CD4^+^ T cells, but only a minor effect on CD8^+^ T cells (53). Thus, the curative effect of blocking CTLA-4 alone cannot be denied in the experiment.

In recent years, some studies have shown that CTLA-4 does not act like traditional immune checkpoints in anti-malignant tumor therapy such as PD-1 but rather activates immunity by clearing regulatory T cells in the tumor microenvironment (54–57). However, our experiments demonstrate that blocking CTLA-4 alone is effective in *E. multilocularis* infection and can indeed reverse T cell exhaustion, but it cannot be ruled out that blocking CTLA-4 has a regulatory T cell clearance effect similar to that in the treatment of malignant tumors, which is also an issue that will be carefully considered in the subsequent studies. This study at least provides a novel target for the improvement and optimization of therapeutic strategies against *E. multilocularis*.

## Data availability statement

The original contributions presented in the study are included in the article/**Supplementary Material**. Further inquiries can be directed to the corresponding author.

## Ethics statement

The studies involving humans were approved by the ethics committee of Affiliated Hospital of Qinghai University (P-SL-2019039). The studies were conducted in accordance with the local legislation and institutional requirements. The participants provided their written informed consent to participate in this study. The animal study was approved by the ethics committee of Affiliated Hospital of Qinghai University (P-SL-2019039). The study was conducted in accordance with the local legislation and institutional requirements.

## Author contributions

YY: Conceptualization, Investigation, Methodology, Resources, Visualization, Writing – original draft, Writing – review & editing. TW: Conceptualization, Project administration, Visualization, Writing – review & editing. BW: Investigation, Methodology, Resources, Validation, Writing – review & editing. SC: Investigation, Methodology, Validation, Writing – review & editing. HF: Conceptualization, Funding acquisition, Project administration, Resources, Supervision, Writing – review & editing.

## Supplementary material

Supplementary Figure 1 Representative H&E staining and Masson trichrome staining of liver sections from alveolar echinococcosis (AE) patients and mice infected with *E. multilocularis*. **(A)** H&E staining of the liver sections from AE patients showed the tissue morphology, and dotted lines were used to delimit the infiltrating margin, liver parenchyma, and lesion. Scale bar = 1,000 μm. **(B)** Masson trichrome staining of the liver sections from AE patients showed liver fibrosis. Collagen fibers appear blue, and yellow arrows indicate the accumulation of collagen fibers. Dotted lines were used to delimit the infiltrating margin, liver parenchyma, and lesion. Scale bar = 1,000 μm. **(C)** H&E staining of the liver sections from mice infected with *E. multilocularis* showed the tissue morphology, and dotted lines were used to delimit the infiltrating margin, liver parenchyma, and lesion. Scale bar = 1,000 μm. **(D)** Masson trichrome staining of the liver sections from mice infected with *E. multilocularis* showed liver fibrosis. Collagen fibers appeared blue, and yellow arrows indicate the accumulation of collagen fibers. Dotted lines are used to delimit the infiltrating margin, liver parenchyma, and lesion. Scale bar = 1,000 μm. IC, inflammatory cells; LP, liver parenchyma.

Supplementary Figure 2 Flow cytometry detection of T cell exhaustion in peripheral blood T cell. **(A)** Representative flow cytometry plots of CD4^+^ CTLA-4^+^ T cells and CD8^+^ CTLA-4^+^ T cells in the peripheral blood of mice infected with *E. multilocularis* or control group. **(B)** Percentage of CD4^+^CTLA-4^+^T cells and CD8^+^CTLA-4^+^T cells in the peripheral blood of mice infected with *E. multilocularis* or control group (*n* = 5). **(C)** Representative flow cytometry plots of TNF-α, IFN-γ, IL-2, Granzyme B, and perforin production by CD4^+^ T cells or CD8^+^ T cells in the peripheral blood of mice infected with *E. multilocularis* that did or did not express CTLA-4. **(D)** Percentage of TNF-α, IFN-γ, IL-2, Granzyme B, and perforin production by CD4^+^ T cells or CD8^+^ T cells in the peripheral blood of mice infected with *E. multilocularis* that did or did not express CTLA-4 (*n* = 5). The data were presented as mean ± SD. **P* < 0.05, ***P* < 0.01, ****P* < 0.001. Con, control; E.m, *E. multilocularis*.
