# Supplementary material for: The expression of CTLA-4 in hepatic alveolar echinococcosis patients and blocking CTLA-4 to reverse T cell exhaustion in Echinococcus multilocularis-infected mice
Source: Front Immunol. 2024 Mar 28;15:1358361. doi: 10.3389/fimmu.2024.1358361 (PMC11007148; doi:10.3389/fimmu.2024.1358361)
Supplement: Supplementary file 1 [file DataSheet_1.docx]

Supplementary Material

The expression of CTLA-4 in hepatic alveolar echinococcosis patients and blocking CTLA-4 to reverse T cell exhaustion in *Echinococcus multilocularis*-infected mice

Yuxuan Yang, Tana Wuren, Binjie Wu, Shilei Cheng, Haining Fan^*^

*** Correspondence:** Haining Fan: [fanhaining@medmail.com.cn](mailto:fanhaining@medmail.com.cn)

# Supplementary Methods

**HE staining and Masson trichrome staining of the liver in patients and mice infected with *E. multiolocularis***

The tissues were washed in phosphate-buffered saline (PBS) and fixed in paraformaldehyde for 48 hours. After fixation, the tissue was embedded in paraffin and cut into 3um sections, dewaxed and hydrated. For HE staining, hematoxylin and eosin were used to show tissue morphology. For Masson staining, by using the Masson trichrome staining kit (Solarbio, Beijing, China), collagen fibers appear blue. After staining, the sections were sealed with neutral gum and observed under an optical microscope.

**Immunohistochemistry analysis of the liver in patients and mice infected with *E. multiolocularis***

The sections were dewaxed, hydrated, and processed for heat-mediated antigen-retrieval by using citric acid buffer. The sections were blocked with goat serum, and then primary antibody (Anti-CTLA4 antibody [CAL49] ,1:500, abcam) were added and incubated overnight at 4 ℃. The next day, the primary antibody was washed with PBS and incubated with the second antibody at room temperature for 1 hour. Visualization was induced by using a diaminobenzidine substrate kit, and sealing with neutral gum. All images were acquired by Tissue FAXS PLUS system (Tissue Gnostics, Australia), and the percentage of CTLA-4 positive cells were analyzed.

**Western blotting analysis of the liver in patients and mice infected with *E. multiolocularis***

The liver tissue from AE patients and mice infected with E. multilocularis were lysed on ice using radioimmunoprecipitation assay buffer containing phenylmethylsulfonyl fluoride, and the lysis was accelerated using a tissue homogenizer. The lysates were centrifuged at 4 °C and 12000 rpm for 20 min. The protein concentration of each group was adjusted to the same level, and boiled in a 95℃ water for 10min, and preserved at −80°C. The samples were separated by electrophoresis of 10% sodium dodecyl sulfate–polyacrylamide gel, after which the proteins were transferred to 0.2μm polyvinylidene fluoride membranes (Merck Millipore, Darmstadt, Germany). Nonspecific antigens were blocked with 5% nonfat milk at room temperature for 1 h, and incubated overnight with primary antibodies (Anti-CTLA4 antibody [CAL49] ,1:1000, abcam). On the second day, TBST solution was used for washing, secondary antibodies of horseradish peroxidase-labelled goat anti-rabbit immunoglobulin G (IgG)were incubated at room temperature for 1h, and bands were exposed using ECL. The greyscale values were measured with ImageJ 1.8.0 software.

**Enzyme‑linked immunosorbent assay of BALB/c splenocytes culture medium**

For in vitro experiments, the cell culture medium was collected and the levels of TNF- α, INF- γ, IL-2, Granzyme B, and Perforin in each group were measured with a commercial ELISA kit (Wuhan, China). For in vivo experiments, mouse serum was collected and measured in the same manner as the vitro experiments.

**Ultrasound assay of the** **lesions in mice livers**

Mice were anesthetized with isoflurane. Small animal ultrasound (ZS3 Exp, mindray, Shenzhen, China) was used to detect lesions located in the liver of mice. The long diameter and short diameter of the ultrasound-detectable were measured respectively.

**Serum ALT and AST Levels**

The serum of mice was collected, and the alanine aminotransferase (ALT) and aspartate aminotransferase (AST) in serum were measured by automatic biochemical analyzer (Chemray 240, Rayto, Shenzhen, China).

# Supplementary Figures and Tables

## Supplementary Figures


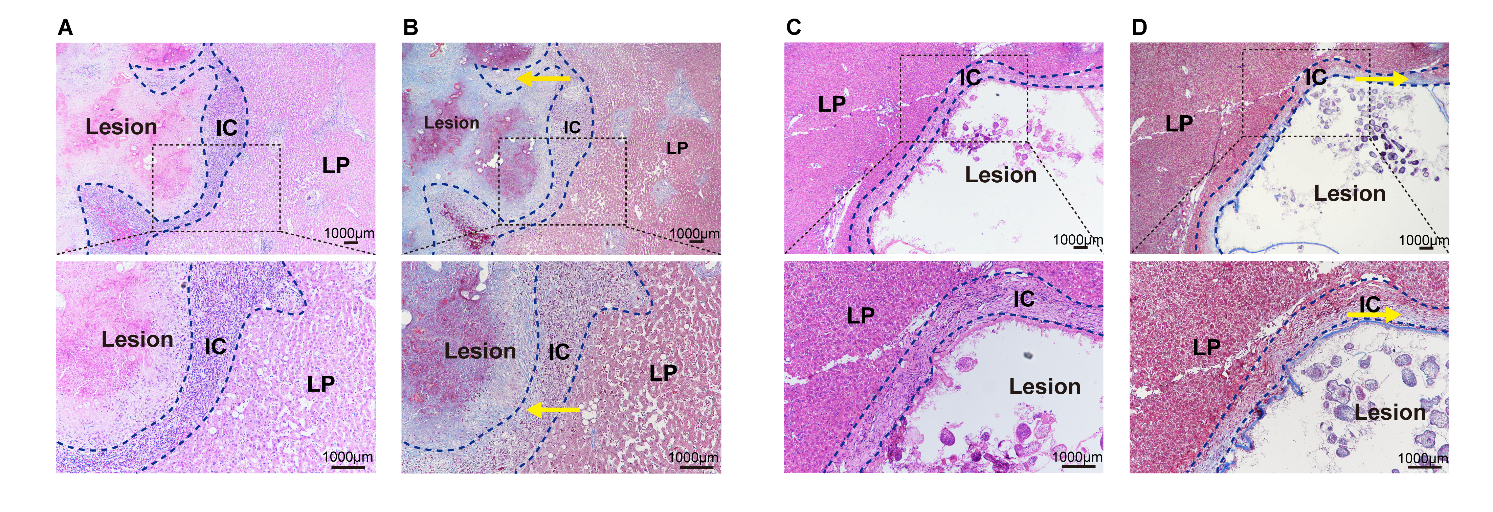


**Supplementary Figure 1.** Representative HE staining and Masson trichrome staining of liver sections from AE patients and mice infected with *E. multilocularis*. **(A)** HE staining of the liver sections from AE patients showed the tissue morphology, and dotted lines were used to delimit the infiltrating margin, liver parenchyma and lesion, Scale bar = 1000 μm. **(B)** Masson trichrome staining of the liver sections from AE patients showed the liver fibrosis. Collagen fibers appear blue, and yellow arrows indicate the accumulation of collagen fibres. Dotted lines were used to delimit the infiltrating margin, liver parenchyma and lesion, Scale bar = 1000 μm. **(C)** HE staining of the liver sections from mice infected with *E. multilocularis* showed the tissue morphology, and dotted lines were used to delimit the infiltrating margin, liver parenchyma and lesion, Scale bar = 1000 μm. **(D)** Masson trichrome staining of the liver sections from mice infected with *E. multilocularis* showed the liver fibrosis. Collagen fibers appeared blue, and yellow arrows indicate the accumulation of collagen fibres. Dotted lines are used to delimit the infiltrating margin, liver parenchyma and lesion, Scale bar = 1000 μm. IC，inflammatory cells; LP，liver parenchyma.


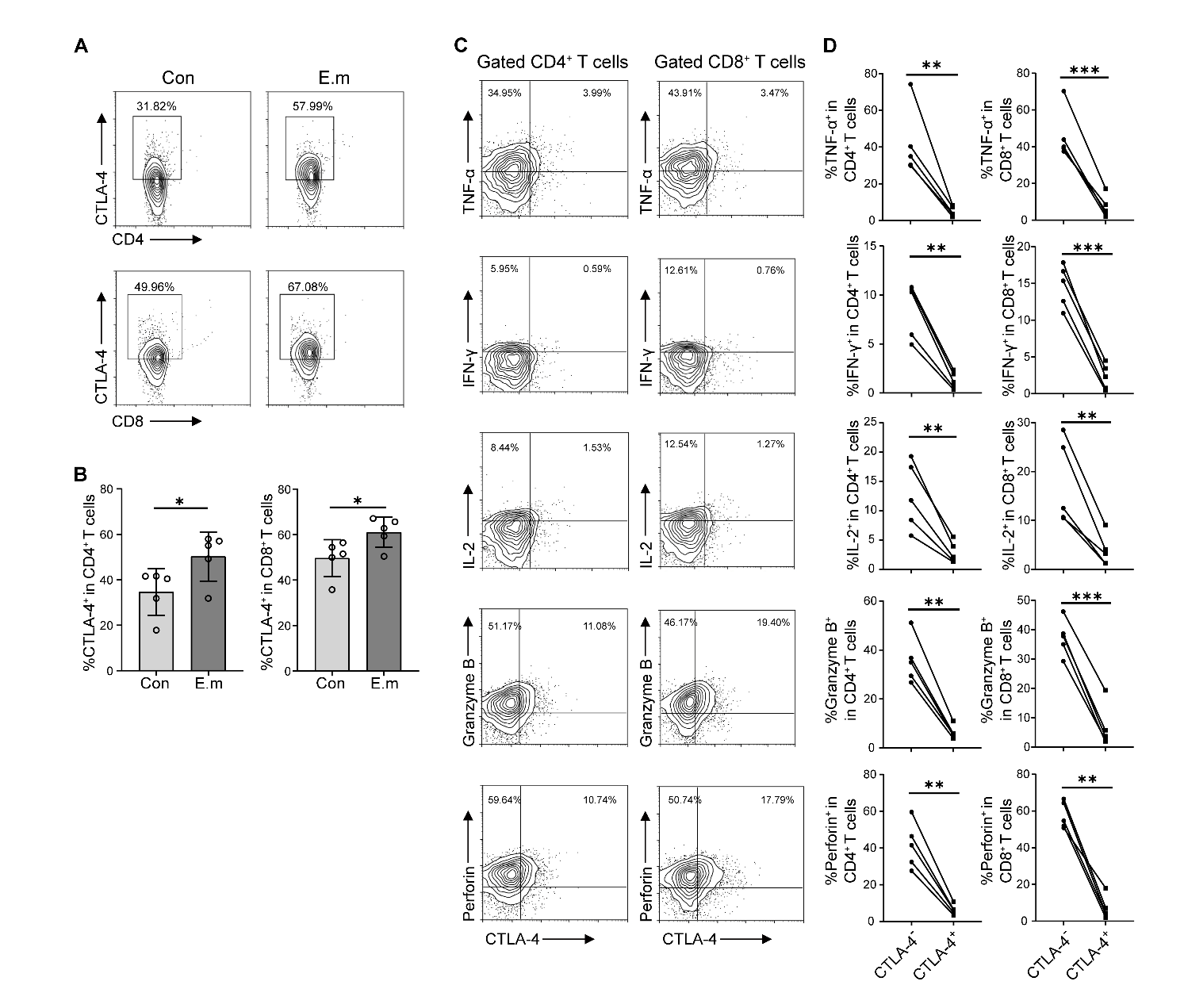


**Supplementary Figure 2.** Flow cytometry detection of T cell exhuastion in peripheral blood T cell. **(A)** Representative flow cytometry plots of CD4^+^ CTLA-4^+^ T cells and CD8^+^ CTLA-4^+^ T cells in the peripheral blood of mice infected with *E. multilocularis* or control group. **(B)** Percentage of CD4^+^ CTLA-4^+^ T cells and CD8^+^CTLA-4^+^T cells in the peripheral blood of mice infected with *E. multilocularis* or control group(n=5). **(C)** Representative flow cytometry plots of TNF-α, IFN-γ, IL-2, Granzyme B and Perforin production by CD4^+^ T cells or CD8^+^ T cells in the peripheral blood of mice infected with *E. multilocularis* that did or did not express CTLA-4. **(D)** Percentage of TNF-α, IFN-γ, IL-2, Granzyme B and Perforin production by CD4^+^ T cells or CD8^+^ T cells in the peripheral blood of mice infected with *E. multilocularis* that did or did not express CTLA-4 (n=5). The data were presented as the mean±SD, * P<0.05，** P<0.01， *** P<0.001. Con, control; E.m, *E. multilocularis*.


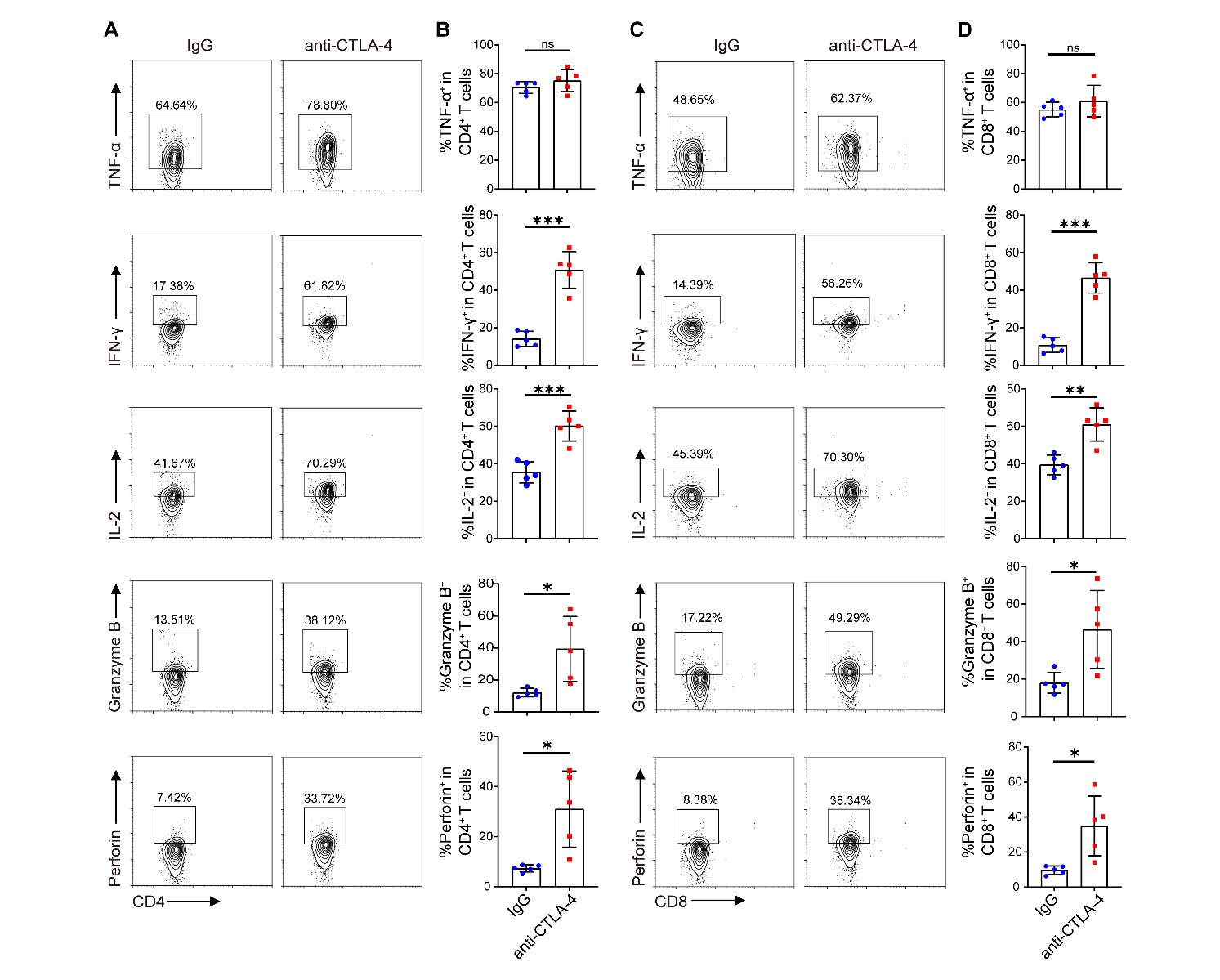


**Supplementary Figure 3.** Blocking CTLA-4 reversed T cell exhaustion in the peripheral blood of mice. **(A)** Representative flow cytometry plots of TNF-α, IFN-γ, IL-2, Granzyme B, and Perforin expression in peripheral blood CD4^+^ T cells of mice treated with anti-CTLA-4 antibody and IgG antibody. **(B)** Percentage of TNF-α, IFN-γ, IL-2, Granzyme B, and Perforin expression in peripheral blood CD4^+^ T cells of mice treated with anti-CTLA-4 antibody and IgG antibody (n=5). **(C)** Representative flow cytometry plots of TNF-α, IFN-γ, IL-2, Granzyme B, and Perforin expression in peripheral blood CD8^+^ T cells of mice treated with anti-CTLA-4 antibody and IgG antibody. **(D)** Percentage of TNF-α, IFN-γ, IL-2, Granzyme B, and Perforin expression in peripheral blood CD8^+^ T cells of mice treated with anti-CTLA-4 antibody and IgG antibody (n=5). The data were presented as the mean±SD, * P<0.05，** P<0.01， *** P<0.001，no significant difference（ns）P>0.05.

**Supplementary Table 1. All antibodies**

| Antibodies | SOURCE | IDENTIFIER |
| --- | --- | --- |
| Anti-CTLA4 antibody [CAL49] | Abcam | Cat: ab237712 |
| Anti-CD4 antibody [EPR6855] | Abcam | Cat: ab133616 |
| Anti-CD4 antibody [EPR19514] | Abcam | Cat: ab183685 |
| Anti-CD8 alpha antibody [EPR21769] | Abcam | Cat: ab217344 |
| Anti-CD8 alpha antibody [EPR22483-288] | Abcam | Cat: ab245118 |
| FITC anti-mouse CD3 Antibody (clone 17A2) | Biolegend | Cat: 100204 |
| APC/Fire™ 750 anti-mouse CD45 Antibody (clone 30-F11) | Biolegend | Cat: 103154 |
| PerCP/Cyanine5.5 anti-mouse CD4 Antibody (clone RM4-5) | Biolegend | Cat: 100540 |
| Brilliant Violet 510™ anti-mouse CD8α Antibody (clone 53-6.7) | Biolegend | Cat: 100752 |
| Brilliant Violet 421™ anti-mouse CD152 Antibody (clone UC10-4B9) | Biolegend | Cat: 106312 |
| PE/Dazzle™ 594 anti-mouse IL-2 Antibody (clone JES6-5H4) | Biolegend | Cat: 503840 |
| APC anti-mouse TNF-α Antibody (clone MP6-XT22) | Biolegend | Cat: 506308 |
| PE anti-mouse IFN-γ Antibody (clone XMG1.2) | Biolegend | Cat: 505808 |
| APC anti-human/mouse Granzyme B Recombinant Antibody(clone QA16A02) | Biolegend | Cat: 372204 |
| PE anti-mouse Perforin Antibody (clone S16009A) | Biolegend | Cat: 154306 |
| In Vivo MAb anti-mouse CD28 (clone 37.51) | BioXCell | Cat: BE0015-1 |
| In Vivo MAb anti-mouse CD3ε (clone 145-2C11) | BioXCell | Cat: BE0001-1 |
| In Vivo Plus anti-mouse CTLA-4 (CD152) (clone9H10) | BioXCell | Cat: BP0131 |
| In Vivo MAb polyclonal Syrian hamster IgG | BioXCell | Cat: BE0087 |
